# Metabolic Profile of Strawberry Fruit Ripened on the Plant Following Treatment With an Ethylene Elicitor or Inhibitor

**DOI:** 10.3389/fpls.2020.00995

**Published:** 2020-07-10

**Authors:** Leticia Reis, Charles F. Forney, Michael Jordan, Kathleen Munro Pennell, Sherry Fillmore, Michelle O. Schemberger, Ricardo A. Ayub

**Affiliations:** ^1^ Laboratório de Biotecnologia Aplicada a Fruticultura, Departamento de Fitotecnia e Fitossanidade, Universidade Estadual de Ponta Grossa, Ponta Grossa, Brazil; ^2^ Kentville Research and Development Centre, Agriculture and Agri-Food Canada, Kentville, NS, Canada

**Keywords:** nonclimacteric, maturation, fruit quality, ethylene, Ethrel^®^, Harvista™, fruit ripening

## Abstract

Strawberry is the most studied nonclimacteric fruit for understanding the role ethylene has in ripening regulation. However, previous studies on the effects of ethylene on strawberry ripening were conducted with detached fruit. Thus, the aim of this work was to determine the effect of ethylene and the ethylene-action inhibitor 1-methylcyclopropene (1-MCP) applied at different developmental stages on important physical-chemical attributes of ripe ‘Albion’ strawberry. Fruit at four developmental stages that remained attached to the plant were dipped in one of three treatment solutions (Ethephon, 1-methylcyclopropene, and water), plus one absolute control that received no dip. Following treatment, when immature fruit were fully red or 24 h after treatment for red-treated fruit, strawberry fruit were assessed for physicochemical properties (mass, length, diameter, firmness, color, titratable acidity, soluble solids, pH, total phenolics, sugar, organic acid, amino acid, and volatile composition). The days following treatment required for fruit to ripen were also recorded. Treatments did not affect the rate of ripening nor fruit color, titratable acidity, pH, soluble solids, total phenolics, sugars, or organic acids of ripe fruit. Ethephon affected fruit mass, diameter, length, firmness, anthocyanins, amino acids, and volatiles, but these effects were dependent on fruit developmental stage at which the treatment was applied. When green fruit were treated with ethephon, ripe fruit had larger diameter and mass. Ethephon treatment of white fruit resulted in ripe fruit having greater anthocyanin content. Treatment of pink fruit resulted in ripe fruit having smaller diameter, length, and mass and greater firmness. Treatment of red fruit with ethephon altered fruit volatile composition, increasing concentrations of ethyl- and acetate-esters, which were reduced by the 1-MCP treatment. Ethephon treatment increased concentrations of 11 of the 19 free amino acids measured in ripe fruit with treatment of green and white fruit having the greatest effect. A total of 41 volatile compounds had significant correlations with 14 amino acids. While ethylene did not stimulate typical ripening of strawberry fruit, it does appear to alter fruit development and metabolism. The physiological effects of ethylene on strawberry fruit appear to depend on the developmental stage of the fruit.

## Highlights

Exogenous ethylene treatment did not alter the rate of strawberry fruit ripening when fruit remained attached to the plant, but depending on the fruit developmental stage, treatments affected fruit growth, free amino acids and volatile metabolism.

## Introduction

The strawberry plant (*Fragaria x ananassa* Duch.) is a crop of high commercial value and world production was greater than 9 million tons in 2016 (http://faostat.fao.org/site/567/default.aspx). The strawberry fruit is composed of dry achenes (true fruit) and the enlarged receptacle (flesh part) that shows high metabolic synchrony during fruit development and ripening (Fait et al., 2008).

Due to low rates of ethylene production and respiration during ripening, strawberry fruit are classified as nonclimacteric (Barry and Giovannoni, 2007; Bapat et al., 2010). However, recent studies have suggested ethylene (Trainotti et al., 2005; Iannetta et al., 2006; Bapat et al., 2010; Villarreal et al., 2010; Merchante et al., 2013; Sun et al., 2013; Lopes et al., 2015) and its interaction with other plant growth regulators such as abscisic acid (Jia et al., 2011; Symons et al., 2012; Jia et al., 2013; Ayub et al., 2016; Jia et al., 2016; Tosetti et al., 2020) and brassinosteroid (Ayub et al., 2018b; Ayub et al., 2018a) may affect strawberry fruit ripening.

Previous studies that used ethylene elicitors or inhibitors have suggested that exogenous ethylene affects some important quality attributes in strawberry fruit, including firmness (Jiang et al., 2001; Villarreal et al., 2008; Villarreal et al., 2009; Villarreal et al., 2016; Elmi et al., 2017), anthocyanin accumulation and phenylalanine ammonia lyase (PAL) activity (Villarreal et al., 2009; Villarreal et al., 2010; Merchante et al., 2013; Sun et al., 2013; Lopes et al., 2015), phenolic compound accumulation (Villarreal et al., 2010; Lopes et al., 2015), organic acids (Merchante et al., 2013; Lopes et al., 2015; Elmi et al., 2017), sugars (Villarreal et al., 2010; Villarreal et al., 2016; Tosetti et al., 2020) and volatile-related genes (Merchante et al., 2013). However, most of these studies have been conducted with immature strawberry fruit following detachment from the plant, which may alter fruit physiology. Absence of literature that evaluates the effect of ethylene on fruit *in situ* is partly due to the unavailability of products that can be applied directly to the fruit, since the ethylene action inhibitor 1-methylcyclopropene (1-MCP) is gaseous. However, the recent development of a new liquid 1-MCP formulation (Harvista™) presents a new opportunity to inhibit ethylene action on fruit still attached to the plant. Therefore, using the liquid ethylene formulation - Ethrel^®^ (an ethylene releasing agent) and the new liquid 1- MCP formulation - Harvista™, the aim of this work was to understand the role of ethylene on normal strawberry ripening on the plant. This was accomplished by measuring the effects of these compounds on the rate of ripening and physicochemical properties of ripe strawberry fruit treated during development and maturation.

## Material and Methods

### Plant Material and Growth Conditions

In 2017, two hundred eight (208) day neutral strawberry plants (*Fragaria × ananassa* cv. Albion) were cultivated in a semi-hydroponic system in a greenhouse at the Kentville Research and Development Centre, Agriculture and Agri-Food Canada (Kentville, Nova Scotia, Canada). Primary or secondary fruit in each of the green, white, pink, and red development stages were characterized and selected daily for treatment. The green stage was characterized as green fruit with developed, separated green achenes and white receptacle color close to the sepals, but still green on the end of the fruit (**Supplementary Figure S1A**). The white stage was defined as fruit with a white receptacle and light green/yellow achenes that were separated from each other at the end of the fruit (**Supplementary Figure S1B**). The pink stage was defined as fruit showing the initial change of color being pink in most of the fruit, but with a white color close to the sepals (**Supplementary Figure S1C**). The red stage was defined as the first-day fruit were observed to have a uniform red color (ripe fruit) (**Supplementary Figure S1D**). For all analyzes four biological replicates of each development stage (obtained from different plants), three treatments (see *Treatment* section) and four technical replicates (see *Experimental Design and Statistical Analysis* section) were used.

### Treatments

Based on the developmental stages described above, ‘Albion’ strawberry fruit attached to the plant were labeled and dipped in one of 3 treatments or received no-dip (absolute control). For the ethylene treatment, whole fruit were dipped for 5 min in a solution of 7 mmol L^−1^ ethephon (Ethrel^®^, 240 g L^-1^ a.i., 2-chloroethylphosphonic acid, which is metabolized by the plant into ethylene), 0.2 ml L^−1^ Tween 20^®^ and 10 ml L^−1^ ethanol (both used as surfactants) that was prepared immediately before use (Villarreal et al., 2010; Sun et al., 2013). For the ethylene inhibitor treatment, whole fruit were submerged for 1 min in a water solution with 1.0 μg L^-1^ aqueous 1-methylcyclopropene (1-MCP, Harvista™ AF-701 formulation, 1.3% a.i, AgroFresh, Inc.). As a control treatment, the whole fruit were submerged for 5 min in a solution of water, 0.2 ml L^−1^ Tween20 and 10 ml L^−1^ ethanol. In addition, fruit were identified at the green stage but were not subjected to a dip to comprise the absolute control. All treated and control fruit were harvested at the red ripe stage. The strawberry fruit treated at the red ripe stage were harvest 24 h after treatment.

### Rate of Ripening

For all fruit treated at the green, white, and pink stages, the number of days from treatment to harvest was recorded to determine the effect of treatment on the rate of ripening. Fruit were harvested at the red ripe stage.

### Physicochemical Fruit Analysis

All fresh fruit were measured for (1) mass, using an analytical balance and expressed in grams (g); (2) diameter and length using a digital caliper and expressed in millimeters (mm); (3) epidermis surface color using a Minolta CR 400 colorimeter and expressed as a* (green/red coordinate: –a indicates green tones and + indicates red color), and hue angle (true color value of the fruit, expressed in degrees), and (4) firmness using a penetrometer with a 6 mm diameter notched tip, expressed in newtons (N). After these measurements, all 12 fruit for each treatment were sliced, frozen in liquid nitrogen and stored as a composite sample in a −80°C ultra low freezer and later analyzed for (5) soluble solids (SS, °Brix), and (6) titratable acidity (%TA, expressed as citric acid equivalents).

### Anthocyanins and Phenolic Compounds

About 6 g of frozen fruit from each composite sample were ground with a mortar and pestle using liquid nitrogen. Approximately 0.5 g of the resultant powder was mixed with 0.7 ml of extraction solvent (methanol/water/trifluoroacetic acid – 70:29:1) in a labeled 2 ml microcentrifuge tube, vortexed for 10 s and sonicated in water for 20 min. The slurry was centrifuged at 10,000*g* at 4°C for 10 min and the supernatant was transferred to a second labelled tube and set aside. Second and third 0.7-ml extractions were conducted on the tissue pellet and the supernatant of the three extractions was pooled. Each treatment was extracted in triplicate.

For anthocyanin measurement, 25 µl of each extracted sample described above were added in triplicate to a 96-well microplate with 275 µl of pH 1.0 buffer (25 ml of 0.2 N KCl and 75 ml of 0.2 N HCl - pH adjusted to 1.0) as well as to a 96-well microplate with pH 4.5 buffer (80 ml of 1M C_2_H_3_NaO_2,_ 50 _ml_ of 1 N HCl and 70 ml of water – pH adjusted to 4.5), according to the pH differential method of Lee et al. (2005). After 5 min, the optical density (OD) at 520 and 700 nm was measured for each plate and then subtracted to determine anthocyanin concentration. The total anthocyanin content was expressed as mg of pelargonidin-3-glucoside (P3g)/100 g fresh mass using the formula: Concentration = (Absorbance × MW × Dilution Factor × 1000)/(є × path length × Sample mass (0.5g)). Where, Absorbance is (A520 nm pH 1.0 - A700 nm pH 1.0) - (A520 nm pH 4.5 - A700 nm pH 4.5); Dilution factor is the total volume (300 µl)/extract volume used (25 µl); The microplate path length (cm) is (4 × Vol)/(π × *d*2), where Vol is in cm^3^ (ml) and *d* is the mean diameter of the well in cm (as listed by the manufacturer). When following this protocol, 300 μl gives a path length of 0.8515 cm. To calculate P3g, an extinction coefficient (є) of 26,900 and molecular weight (MW) of 433.389 g/mol was used.

For phenolic compound measurement, 25 µl of extract from each sample was added in triplicate to 250 µl milli-Q water and 50 µl prepared Folin-Ciocalteu reagent: Water (1:2 (v:v)) in a 96-well microplate, according to the Folin-Ciocalteu method (Singleton et al., 1965). A gallic acid (0, 50, 75, 100, 150, 200, and 250 mg/L) standard curve was done in triplicate for each microplate and used to quantify total phenolics. After 5 min of agitation, 12 µl of a saturated Na-carbonate solution was added to each microplate well, followed by additional agitation. After a 1-h rest in the dark at room temperature, the OD at 750 nm was measured. Total phenolic compounds were calculated as gallic acid equivalents/100 g sample using the absorbance and the regression equation for the standards, where concentration = {[((absorbance - intercept)/slope)/1000]/dilution factor} × 100.

### Sugars, Organic Acids and Amino Acids

About 20 g of frozen fruit tissue from each treatment were thawed, crushed, and centrifuged in 50-ml tubes for 20 min. The supernatant strawberry juice was filtered through a polyethylene membrane of 0.45-µm pore size (Milli- pore Corp., Bedford, MA, USA) and collected directly in three different vials (2 × 2 ml) for sugar, organic acid, and amino acid analysis.

The juice was analyzed for sugar composition using an Agilent UHPLC 1290 Infinity II System equipped with a 1260 Infinity II Refractive Index Detector G7162A held at 40°C. Using a 1290 Infinity II Multisampler G7116B, a 20-µl sample of juice was injected onto a BioRad, Aminex HPX-87P column with a guard held at 85°C and with a flow rate of 0.6 ml/min of 100% Nanopure water. The water was vacuum filtered with a 0.22 µ GV Durapore membrane filter and sonicated for about 10 min prior to use. The sugars sucrose, glucose, and fructose were identified by retention times, which were 10.11, 11.12, and 18.8 min respectively. All sugars were identified and quantified using external standards. Quantification of these compounds was carried out by calibration curves constructed with three independent sets of dilutions of standard compounds.

For organic acid analysis, the juice was analyzed using an Agilent UHPLC 1290 Infinity II System equipped with a diode array detector. A 20-µl sample of juice was injected onto a Phenomenex, Kinetex 2.6 µ 100A, 150 × 4.6 mm column with a guard held at 30°C and with a flow rate of 0.5 ml/min of 0.01M H_2_SO_4_, pH 2.5 that had been vacuum filtered with a 0.22 µ RC filter and sonicated for about 10 min. Malic, shikimic, citric and succinic acids were identified by retention times which were 4.00, 5.93, 6.79, and 20.4 min respectively. All organic acids were identified and quantified using external standards.

For amino acid analysis, the juice was analyzed using an Agilent UHPLC 1290 Infinity II System equipped with a fluorescence detector. A 40-µl sample of juice was injected onto an Agilent, Infinity Lab Poroshell HPH-C18 column with a guard held at 40°C with a flow rate of 0.42 ml/min. The mobile phase consisted of a linear gradient of 98% phase A decreasing to 43% after 13.4 min, then decreasing to 0% and returning to 98% after 15.8 min. Phase A consisted of 10 mM Na_2_HPO_4_ and 10 mM Na_2_B_4_O_7_ at pH 8.2 that was vacuum filtered with a 0.22µ RC filter and sonicated for about 10 min and phase B consisted of acetonitrile: methanol: nanopure water 45:45:10 (v:v:v). Detection was carried out using fluorescence, with excitation at 340 nm and emission at 450 nm for the first 10.3 min followed by excitation at 260 nm and emission at 315 nm. The amino acids alanine, arginine, asparagine, aspartate, cystine, glutamate, glutamine, glycine, histidine, isoleucine, leucine, lysine, phenylalanine, serine, threonine, tryptophan, tyrosine, and valine were identified and quantified using external standards.

### Volatile Compounds

For each treatment, 5 g of composite frozen sample (−80 C) were blended with 100 g of a saturated salt solution (NaCl) for 1 min at a setting of 4 using a Kinematica, model MB 800 Laboratory Mixer (Kinematica AG, Luzern, Switzerland). A 10-g sample of the homogeneate was placed in a 20-ml headspace vial, capped and 5 µl of an internal standard mix (9.8 mg L^-1^ ethyl acetate-d8; 10.0 mg L^-1^ benzaldehyde-d6; 10.2 mg L^-1^ 2-phenyl-d5-ethanol; 8.2 mg L^-1^ 2-hexanone-d5) was added using a MultiPurpose Sampler (Gerstel, Linthicum, MD, USA). The volatile compounds were extracted and analyzed by solid phase micro extraction- Two-Dimensional-gas chromatography–time of flight mass spectrometer (SPME-2DGC–TOFMS) using a Pegasus 4D GCxGC-TOFMS system (LECO, St. Joseph, MI, USA). Vials were incubated at 50°C for 300 s and then a divinylbenzene/carboxen/polydimethylsiloxane SPME fiber (Supelco Analytical, Bellefonte, PA, USA) was exposed to the headspace for 900 s with agitation (on for 60 s; off for 1 s). The fiber was desorbed at 250°C for 15 min. The injector was operated at 250°C in the split mode for 1 min at a split of 1:20. Helium was used as the carrier gas at a flow rate of 1.4 ml/min. For GCxGC analysis, a quad-jet dual stage thermal modulator was mounted in an Agilent 7890 GC gas chromatograph equipped with a secondary oven. Liquid nitrogen was used for cooling the cold jet lines. The first-dimension (1D) column was a polar Stabilwax^®^ (30 m × 0.25 mm × 0.25 µm), and the second dimension (2D) column was a mid-polar Rxi^®^-5Sil MS (0.30 m × 0.25 mm × 0.25 µm). The optimized 1D GC oven temperature was initially set at 50°C for 0.20 min, before increasing at 10.3°C/min to 220°C. The temperature offset for the secondary oven was 44°C. The modulation period (PM) was 1.1 s, with a hot pulse time of 0.33 s on each jet. The transfer line was held at 250°C. The TOF-MS was operated in electron ionization (EI) mode at 70 eV, with an acquisition mass range of 35–300 amu, an acquisition rate of 200 Hz, and a detector voltage of 1500 V with an optimized voltage of 200 V. The ion source was heated to 250°C. Daily mass calibration and tuning were performed using perfluorotributylamine (PFTBA). An acquisition delay of 100 s was applied.

The chemical identification of the peaks was determined based on the retention index and correspondence of the mass spectrum with the National Institute of Standards and Technology (NIST) Mass Spectral virtual Library (ChemSW, Fairfield, CA, USA) and possible identifications were confirmed with known standards when available. To calculate total ion counts (TIC) of each compound the LECO APEX data deconvolution/processing routine was used. Area counts were normalized against the internal standard 2-hexanone-d5. Analysis of Variance (ANOVA) and PCA (principal component analysis) were performed on normalized area counts of the volatile compounds that had a relative abundance >0.05%.

To explore relationships between fruit volatiles and free amino acids, correlations were calculated. Using PCA to conduct data reduction analysis, a series of PCA’s were conducted to identify the most important relationships among the amino acids and volatiles. This was achieved by first identifying amino acids and volatiles that were most affected by treatments through separate PCA’s. Additional multivariate analysis was conducted with these volatiles and amino acids to identify amino acids that correlated with volatile compounds.

### Experimental Design and Statistical Analysis

The experiment was carried out in a randomized block design in a 3 × 4 factorial with 1 absolute control. The treatments were composed of three dip solutions [Harvista (1-MCP), Ethephon, and water] applied at four development stages (green, white, pink, and red), plus an absolute control that received no dip. Each treatment was performed in four replicates (blocks) with each replication comprised of four plants. From these four plants, 12 strawberry fruit were treated, harvested and assessed when red ripe. These 12 fruit were combined to form composite samples for chemical analysis. The data collected after harvest of ripe fruit was analyzed using ANOVA with orthogonal contrasts, and PCA through Genstat 16 (VSN International, 2013) software.

## Results

### Ethylene Effects on Rate of Ripening

The rate of ‘Albion’ strawberry fruit ripening measured as red color development was not affected by treatment with ethephon or Harvista regardless of the development stage at which it was applied (**Table 1**). The time from treatment until fruit was red ripe averaged about 13 days for fruit treated at the green stage, 6 days for fruit treated at the white stage and 4 days for fruit treated at the pink stage.

**Table 1:** Comparison of rate of ripening (days to red ripe), and mass (g), diameter (mm), length (mm) and firmness (N) of ripe ‘Albion’ strawberry fruit treated with Harvista (1-MCP), Ethephon or water at four development stages (Green, White, Pink and Red) plus an absolute control (No Dip).

Stage	Treatment	Days to red ripe	Mass (g)	Diameter (mm)	Length (mm)	Firmness (N)
–	NoDip	13.0	13.2	289	347	4.19
Green	1MCP	12.9	11.9	279	332	4.32
	Ethephon	13.5	14.1	295	341	4.49
	Water	11.4	12.2	283	327	4.81
White	1-MCP	5.3	12.9	291	336	4.50
	Ethephon	5.8	13.3	297	331	4.35
	Water	5.5	13.9	296	342	4.41
Pink	1-MCP	3.8	13.1	294	343	4.46
	Ethephon	3.4	10.7	270	308	5.65
	Water	4.3	12.4	287	327	4.20
Red	1-MCP	–	12.2	286	333	4.36
	Ethephon	–	12.4	283	332	4.48
	Water	–	12.6	283	351	4.04
						
Grand Mean		–	12.7	287	335	4.48
SEM		0.31	0.49	4.0	7.0	0.23
F probability						
Main effects	Stage	<0.001	0.018	0.019	NS	0.083
	Dips	NS	NS	NS	NS	0.048
	Stage x Dips	0.002	0.002	0.005	0.023	0.008
Contrasts	1-MCP vs Ethephon	NS	NS	NS	NS	0.045
	G,W vs P,R x 1-MCP vs Ethephon	NS	0.002	<0.001	0.047	0.054
	G vs W x 1-MCP vs Ethephon	NS	0.064	NS	NS	NS
	P vs R x 1-MCP vs Ethephon	NS	0.010	0.023	0.020	0.024

NS, No significant difference (P > 0.10).

### Ethylene Effects on Strawberry Fruit Size and Firmness

Size and firmness of fruit receiving no treatment (No Dip control) were not significantly different than the average of the factorial treatments, but significant interactions between development stages and treatments were observed (**Table 1**). Green fruit treated with Ethephon had 16% and 13.5% greater mass and 5.4% and 4.0% larger diameter when ripe than 1-MCP or water treated fruit, respectively. In contrast, pink fruit treated with ethephon resulted in ripe fruit having 22.5%, 16.2%, and 23.7% less mass, and 9%, 6.3%, and 7.0% smaller diameter and 11.4%, 6.2%, and 12.7% shorter length than fruit treated with 1-MCP, water or NoDip, respectively. No differences in mass, diameter or length were observed for any ripe fruit that were treated at the white or red development stage (**Table 1**). In addition, pink fruit treated with Ethephon were 21.0%, 25.7%, and 25.8% firmer when ripe than those treated with 1-MCP, water or No Dip, respectively. Ethylene or 1-MCP treatments did not affect firmness of strawberry fruit treated at the green, white or red development stages (**Table 1**).

### Ethylene Effects on Anthocyanins, Phenolics and Color Development

Total anthocyanin and phenolic content increased as strawberry fruit ripen. In this study, total anthocyanins, total phenolics and red color formation of the No Dip control fruit was not significantly different than the average of the factorial treatments (**Table 2**). Ethephon and 1-MCP treatments also did not affect the color of ripe fruit, measured as hue angle and a* value nor fruit total phenolic content (**Table 2**). However, while the total anthocyanin content of fruit treated at the green, pink and red development stages was not affected by treatments, total anthocyanin content in ripe fruit that were treated with ethephon at the white development stage were 32.7%, 25.0%, and 22.8% greater than those treated with 1-MCP, water, or the No Dip control, respectively.

**Table 2:** Total anthocyanins (mg/100 g), phenolic compound content (mg of GA/100g) and fruit color expressed as a* (red color) and Hue angle (*h*) in ripe ‘Albion’ strawberry fruit treated with Harvista (1-MCP), Ethephon or water plus an absolute control (No Dip) at four development stages (Green, White, Pink and Red).

Stage	Treatment	Anthocyanins (mg P3g eq/100 g)	Phenolic compounds (mg GA eq/100 g)	Color	
				a*	*h*
–	NoDip	203	34.6	29.5	25.3
Green	1-MCP	202	32.1	29.6	26.7
	Ethephon	222	30.4	29.4	26.3
	Water	205	31.1	28.9	26.2
White	1-MCP	177	32.5	29.9	28.1
	Ethephon	263	33.1	28.1	25.9
	Water	197	33.3	31.0	28.1
Pink	1-MCP	221	33.2	29.5	27.4
	Ethephon	208	32.6	28.9	25.8
	Water	210	31.5	30.9	27.6
Red	1-MCP	234	31.6	30.4	27.6
	Ethephon	225	34.6	30.0	29.4
	Water	220	33.1	30.5	27.7
					
Grand Mean		214	32.6	29.7	27.1
SEM		15.0	1.46	0.68	0.92
F probability					
Main effects	Stage	NS	NS	NS	NS
	Dips	0.082	NS	NS	NS
	Stage x Dips	0.044	NS	NS	NS
Contrasts	1-MCP vs Ethephon	0.056	NS	NS	NS
	G,W vs P,R x 1-MCP vs Ethephon	0.005	NS	NS	NS
	G vs W x 1-MCP vs Ethephon	0.034	NS	NS	NS
	P vs R x 1-MCP vs Ethephon	NS	NS	NS	0.076

NS, No significant difference (P > 0.10).

### Ethylene Effects on Sugar and Organic Acid Content

Sugar accumulation and loss of acidity are associated with strawberry fruit ripening. Ethephon and 1-MCP treatments did not affect sugar concentration or SS content of ripe fruit following treatment at different ripeness stages (**Table 3**). In ‘Albion’ strawberry fruit, glucose and fructose were the most abundant sugars in ripe fruit subjected to all treatments, comprising 39.3% and 43.3% of total sugars, respectively. Sucrose comprised only 17% of the total sugars suggesting a high rate of sucrose hydrolysis in the ripe fruit. The soluble solids content of ripe ‘Albion’ strawberry fruit, which has traditionaly been used to estimate fruit sugar content, averaged 6.9 ± 0.30°Brix.

**Table 3:** Comparison of soluble solids (°Brix) and concentration of sucrose, glucose, fructose and total sugars (mg/ml) in juice of ripe ‘Albion’ strawberry fruit treated with Harvista (1-MCP), Ethephon or water at four development stages (Green, White, Pink and Red) plus an absolute control (No Dip).

Stage	Dip treatment	Soluble solids	Sugar concentration (mg/ml)			
			Sucrose	Glucose	Fructose	Total Sugars
–	NoDip	6.58	2.19	4.52	4.94	11.7
Green	1-MCP	7.53	1.74	3.98	4.36	10.1
	Ethephon	6.91	1.78	3.62	4.08	9.47
	Water	7.50	1.84	3.84	4.24	9.92
White	1-MCP	6.63	1.64	4.03	4.42	10.1
	Ethephon	6.50	1.64	4.33	4.81	10.8
	Water	6.42	2.21	4.54	4.96	11.7
Pink	1-MCP	7.06	1.54	3.85	4.25	9.64
	Ethephon	6.84	1.56	4.13	4.57	10.3
	Water	7.59	1.57	4.18	4.65	10.4
Red	1-MCP	7.01	1.83	4.67	5.10	12.2
	Ethephon	6.48	1.87	4.15	4.60	10.6
	Water	6.66	2.06	4.36	4.79	11.2
						
Grand Mean		6.90	1.80	4.17	4.60	10.6
SEM		0.304	0.234	0.250	0.275	0.756
F probability						
Main effects	Stage	0.009	NS	0.033	0.050	0.076
	Dips	NS	NS	NS	NS	NS
	Stage x Dips	NS	NS	NS	NS	NS
Contrasts	G vs W	0.003	NS	0.023	0.032	NS
	P vs R	0.079	0.066	NS	NS	0.054

NS, No significant difference (P > 0.10).

Similarly, no treatment effect on organic acid content or titratable acids (TA%) was observed in ripe fruit that had been treated at different ripeness stages (**Table 4**). As expected, citric acid was the predominant acid comprising 69.7% of total acids for all ripe fruit treated at different ripeness stages, and was followed by malic acid, which was responsible for 23.9% of the total acids. Other organic acids measured included succinic and shikimic acids that were observed in small amounts and accounted for 7.9% and 0.5% of the total acids respectively. Values of TA averaged 1.05% ± 0.03% citric acid equivalents.

**Table 4:** Comparison of titratable acids (TA%) and concentration of citric, malic, shikimic, succinic and total organic acids (ug/ml) in juice of ripe ‘Albion’ strawberry fruit treated with Harvista (1-MCP), Ethephon or water at four development stages (Green, White, Pink and Red) plus an absolute control (No Dip).

Stage	Treatment	TA	Organic acid concentration (ug/mL)				
			Citric acid	Malic acid	Succinic acid	Shikimic acid	Total acids
–	NoDip	1.03	2410	850	360	18.2	3350
Green	1-MCP	1.05	2220	799	324	17.2	3420
	Ethephon	1.00	2230	834	335	18.6	3400
	Water	1.13	2270	783	331	16.9	3410
White	1-MCP	1.04	2350	770	270	16.4	3450
	Ethephon	1.08	2320	802	307	17.0	3260
	Water	1.05	2290	709	236	19.6	3190
Pink	1-MCP	1.03	2090	772	316	16.9	3520
	Ethephon	1.07	2360	835	311	14.9	3690
	Water	0.953	2450	877	347	19.1	3490
Red	1-MCP	1.06	2360	839	278	19.3	3620
	Ethephon	1.12	2430	834	333	19.0	3710
	Water	1.06	2530	868	288	19.8	3630
							
Grand Mean		1.05	2330	813	311	17.9	3470
SEM		0.032	178	51.7	41.3	2.22	204
F probability							
Main effects	Stage	NS	NS	NS	NS	NS	0.014
	Dips	NS	NS	NS	NS	NS	NS
	Stage x Dips	NS	NS	NS	NS	NS	NS
Contrasts	G,W vs P,R	NS	NS	0.072	NS	NS	NS
	P vs R	0.023	NS	NS	NS	NS	0.002

NS, No significant difference (P > 0.10).

### Ethylene Effects on Amino Acid Metabolism

In ‘Albion’ strawberry fruit subjected to all treatments, asparagine, glutamine and alanine were the most abundant free amino acids, comprising 40%, 18.7%, and 13% of total amino acids, respectively, followed by glutamate, serine and aspartate that comprised 8.3%, 7.2%, and 6.3% of total amino acids, respectively (**Supplementary Table S1**). The other 13 amino acids identified were observed in lower concentrations (Data not shown). The No Dip control was not significantly different than the average of the factorial treatments, but significant effects of the dip treatments were observed (**Supplementary Table S1**). The ethephon treatment increased the concentration of alanine, arginine, glutamine, glycine, histidine, isoleucine, phenylalanine, threonine, tryptophan, tyrosine, and valine in ripe fruit (**Supplementary Table S1**).

**Figure 1:**
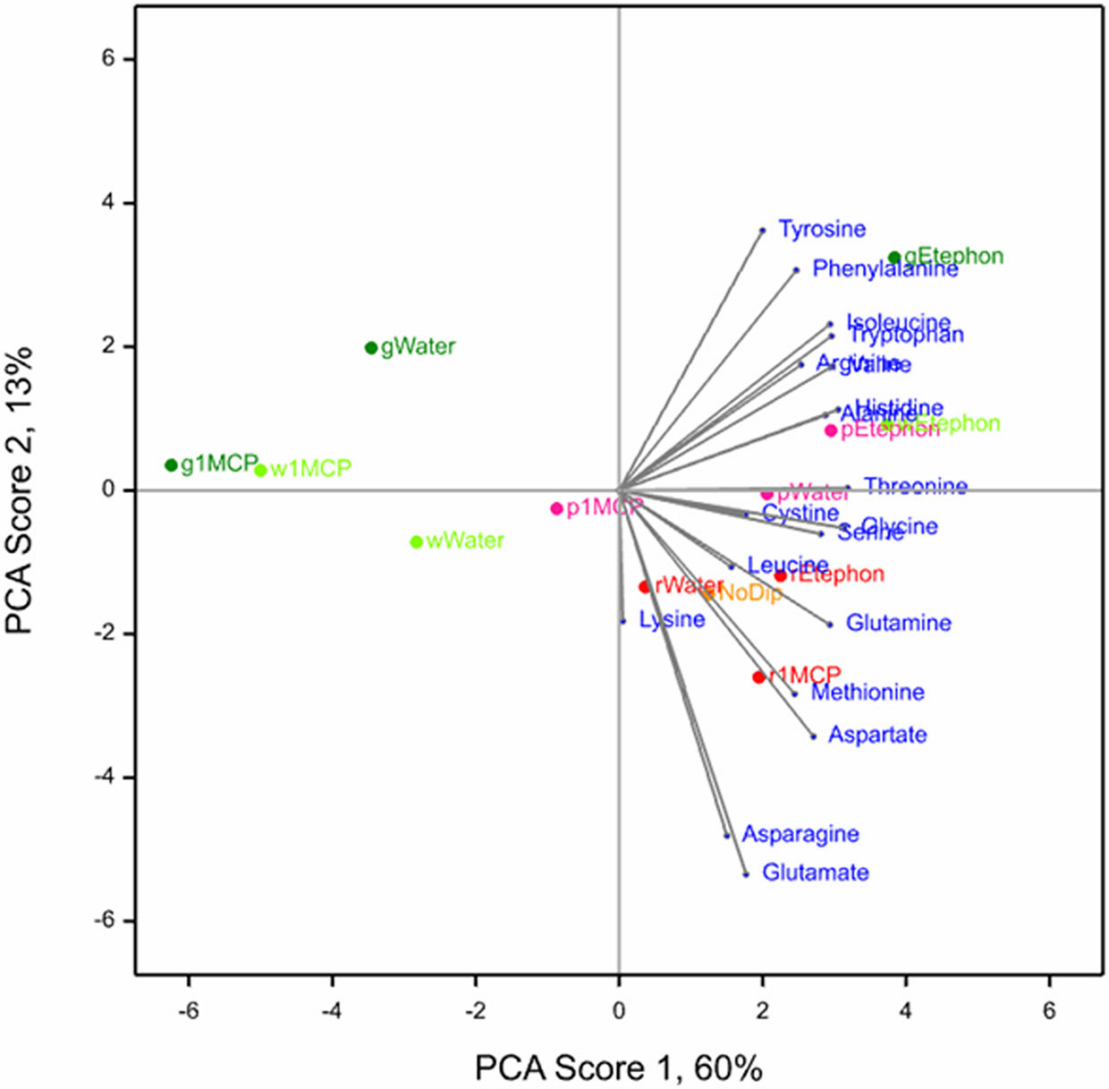
Biplot of the first two scores of the PCA for 19 amino acids observed in ripe ‘Albion’ strawberry fruit treated with Ethephon, 1-MCP or water plus an absolute control (No Dip) at four development stages (Green, White, Pink and Red).

PCA was conducted to observe the interactive effects of treatments and development stages on the 19 amino acids in ripe ‘Albion’ fruit. The biplot of the first 2 scores is shown in **Figure 1**. PC1 Score accounted for 60% and PC2 Score accounted for 13% of the variance of the data. Together they represent more than 70% of the variance.

The PC1 score showed that ethephon treatment was associated with higher concentrations of all amino acids, especially in fruit that were treated at the green and white development stages. The 1-MPC treatment appeared to negate the ethephon effect, which was most apparent in green and white treated fruit and least in the red treated fruit. The amino acids tyrosine, phenylalanine, and isoleucine, and to a lesser extent tryptophan, arginine, valine, histidine and alanine, were associated with fruit treated with ethephon at the green stage and to a lesser extent the white and pink development stages. The amino acids glutamine, asparagine, aspartate and methionine were associated with the red development stage regardless of treatment, including the NoDip absolute control fruit (**Figure 1**).

### Ethylene Effects on Volatile Metabolism

A total of 852 volatile compounds were identified in the headspace of homogenized ripe strawberry fruit (**Supplementary Table S2**), but of these compounds, 81 had a relative abundance >0.05% and these compounds comprised over 95% of the normalized volatile area counts (**Supplementary Table S3**). These 81 compounds were comprised of 17 aldehydes, 18 esters (12 straight-chain, 6 branched), 16 monoterpenoids, 9 ketones, 8 sesquiterpenoids, 4 alcohols, 3 hydrocarbons, 2 furans, 2 lactones, 1 acid, and 1 norisoprenoid. Significant effects (P < 0.1) of stage of treatment and dip treatment were observed in 41 and 22 of these compounds, respectively and 18 had a significant interaction (**Table 5**).

**Table 5:** Volatile compounds of ripe ‘Albion’ strawberry fruit treated with Ethephon, Harvista (1-MCP) or water at four development stages (Green, White, Pink and Red) plus an absolute control (No Dip) ranked by relative abundance.

Compound*	Mean Normalized Area counts	SEM**	% Abundance	Significance (F prob)			
				No dip	Ripeness Stage	Dip Treatment	Ripeness x Dip
d.f.				1	3	2	6
**Grand Total**	**2938**	**256.8**	**100.000**	**ns**	**<0.001**	**0.009**	**<0.001**
(*E*)-hex-2-enal	612.20	86.59	20.84	ns	0.047	.	ns
methyl butanoate	442.90	42.12	15.08	ns	0.011	.	0.023
hexanal	279.20	36.34	9.50	ns	ns	.	ns
3,7-dimethylocta-1,6-dien-3-ol	170.80	28.35	5.81	ns	<0.001	.	ns
ethyl butanoate	147.50	47.14	5.02	0.031	<0.001	<0.001	<0.001
3,7,11-trimethyldodeca-1,6,10-trien-3-ol	125.20	22.80	4.26	ns	ns	0.053	0.019
(E)-hex-3-enal	90.89	23.76	3.09	ns	ns	.	ns
methyl hexanoate	79.36	13.54	2.70	ns	<0.001	.	0.007
methyl acetate	75.34	7.51	2.56	ns	0.054	.	0.056
ethyl acetate	69.59	29.58	2.37	0.029	<0.001	<0.001	<0.001
[(Z)-hex-2-enyl] acetate	67.55	12.12	2.30	ns	<0.001	0.046	ns
propan-2-one	64.26	8.55	2.19	ns	ns	.	ns
(2E,4E)-hexa-2,4-dienal	45.69	6.93	1.56	ns	ns	.	ns
(E)-hex-2-enal	35.11	70.06	1.20	ns	ns	.	ns
5-hexyloxolan-2-one	33.51	11.13	1.14	ns	<0.001	.	ns
butyl acetate	32.00	10.24	1.09	ns	<0.001	<0.001	<0.001
propan-2-yl butanoate	28.92	28.80	0.98	ns	ns	.	ns
2-(4-methylcyclohex-3-en-1-yl)propan-2-ol	26.76	8.27	0.91	ns	0.031	.	ns
hexyl acetate	19.91	4.16	0.68	ns	<0.001	0.04	ns
heptan-2-one	18.65	5.05	0.64	ns	<0.001	.	0.043
pentan-2-one	18.56	9.57	0.63	0.001	ns	.	ns
4-methylpentan-2-one	15.82	3.41	0.54	ns	ns	.	ns
butyl butanoate	15.46	12.38	0.53	ns	<0.001	0.058	0.052
nonanal	11.88	3.55	0.40	ns	ns	0.034	ns
3-methylbutanal	11.07	16.26	0.38	ns	ns	0.095	ns
ethyl hexanoate	10.34	4.28	0.35	ns	<0.001	0.004	<0.001
2-methyl-6-methylideneocta-1,7-diene	9.71	2.15	0.33	ns	<0.001	.	ns
methyl 3-methylbutanoate	9.59	2.83	0.33	ns	ns	.	ns
2-[(2S,5S)-5-ethenyl-5-methyloxolan-2-yl]propan-2-ol	9.26	1.34	0.32	ns	0.053	.	0.033
2-ethylhexan-1-ol	8.93	5.85	0.30	ns	ns	0.065	ns
6,6-dimethyl-2-methylidenebicyclo[3.1.1]heptane	8.86	6.96	0.30	ns	0.043	.	ns
3-methylbutyl acetate	8.77	1.95	0.30	ns	<0.001	0.001	<0.001
3,7,11-trimethyldodeca-1,6,10-trien-3-ol	8.71	15.86	0.30	ns	ns	.	ns
1-methyl-4-propan-2-ylidenecyclohexene	8.39	3.16	0.29	ns	0.017	.	ns
(3E,6E)-3,7,11-trimethyldodeca-1,3,6,10-tetraene	8.20	1.02	0.28	ns	<0.001	.	0.095
propan-2-yl acetate	7.80	1.40	0.27	ns	<0.001	.	0.001
octanal	6.53	1.58	0.22	0.037	ns	0.074	ns
1-methyl-4-prop-1-en-2-ylcyclohexene	6.19	2.76	0.21	ns	ns	.	ns
5-octyloxolan-2-one	6.03	1.72	0.21	ns	<0.001	.	ns
hexanoic acid	5.95	1.08	0.20	ns	<0.001	.	ns
1,2,4-trimethyl-3-nitrobicyclo[3.3.1]nonan-9-one	5.95	2.78	0.20	ns	0.021	.	ns
(E)-hex-3-enal	5.75	12.54	0.20	ns	ns	.	ns
2-ethylfuran	5.58	2.06	0.19	ns	ns	0.098	ns
hexane	5.47	12.62	0.19	0.065	ns	.	ns
pent-1-en-3-one	5.40	0.82	0.18	0.07	ns	.	ns
heptanal	5.18	4.25	0.18	ns	ns	.	ns
6-methylhept-5-en-2-one	5.11	0.49	0.17	0.054	<0.001	.	ns
(E)-hept-2-enal	5.05	2.25	0.17	ns	0.074	.	ns
decanal	4.47	1.74	0.15	ns	ns	.	0.029
2-[(2S,5S)-5-ethenyl-5-methyloxolan-2-yl]propan-2-ol	4.37	0.99	0.15	ns	ns	.	ns
ethyl 3-methylbutanoate	4.37	5.01	0.15	ns	<0.001	.	ns
pentanal	3.81	6.19	0.13	ns	ns	.	ns
(E)-4-(2,6,6-trimethylcyclohex-2-en-1-yl)but-3-en-2-one	3.75	0.91	0.13	ns	ns	.	ns
(3Z)-3,7-dimethylocta-1,3,7-triene	3.65	1.19	0.12	ns	0.017	.	ns
(E)-pent-2-enal	3.37	0.99	0.12	ns	ns	0.079	ns
oct-1-en-3-one	3.26	1.08	0.11	ns	ns	0.076	ns
[(Z)-hex-3-enyl] acetate	3.17	1.22	0.11	ns	0.049	.	ns
3,7,11-trimethyldodeca-1,6,10-trien-3-ol	3.04	10.96	0.10	ns	ns	.	ns
(3S,6S)-2,2,6-trimethyl-6-[(1S)-4-methylcyclohex-3-en-1-yl]oxan-3-ol	2.94	1.62	0.10	ns	ns	.	ns
1-methyl-4-prop-1-en-2-ylcyclohexene	2.93	2.68	0.10	ns	ns	0.048	ns
2-ethenyl-2,6,6-trimethyloxane	2.92	1.14	0.10	ns	0.067	.	ns
(Z)-β-Ocimene(3Z)-3,7-dimethylocta-1,3,6-triene	2.87	1.45	0.10	ns	ns	.	ns
(E)-oct-2-enal	2.78	0.67	0.10	ns	ns	0.052	ns
benzaldehyde	2.77	1.84	0.09	ns	ns	.	ns
Unknown 1	2.73	1.02	0.09	0.081	ns	.	ns
butan-2-one	2.65	0.69	0.09	ns	<0.001	.	0.011
(Z)-hept-2-enal	2.58	1.98	0.09	ns	ns	.	ns
benzyl acetate	2.45	0.43	0.08	ns	<0.001	<0.001	0.001
(3E,6E)-3,7,11-trimethyldodeca-1,3,6,10-tetraene	2.34	0.37	0.08	ns	<0.001	0.099	ns
4-methoxy-2,5-dimethylfuran-3-one	2.16	0.86	0.07	ns	0.006	0.098	ns
1-methoxy-2-methylpropan-2-ol	2.12	0.60	0.07	ns	ns	.	ns
2-(4-methylcyclohex-3-en-1-yl)propanal	1.97	0.28	0.07	ns	0.058	.	ns
(2E)-3,7-dimethylocta-2,6-dien-1-ol	1.94	0.86	0.07	0.094	0.051	.	0.095
hexan-1-ol	1.93	0.40	0.07	ns	ns	.	ns
5-methylhexa-1,2-diene	1.84	3.63	0.06	0.042	ns	.	ns
(2E)-3,7-dimethylocta-2,6-dien-1-ol	1.75	1.22	0.06	ns	ns	.	ns
(3Z,6E)-3,7,11-trimethyldodeca-1,3,6,10-tetraene	1.74	0.25	0.06	ns	<0.001	.	ns
(2R)-2-ethenyl-2,6,6-trimethyloxane	1.70	0.50	0.06	ns	ns	.	ns
octyl butanoate	1.59	0.75	0.05	ns	0.003	.	ns
1-methyl-4-[(2E)-6-methylhepta-2,5-dien-2-yl]cyclohexene	1.56	0.27	0.05	ns	<0.001	.	ns
octan-1-ol	1.55	0.40	0.05	ns	ns	.	ns
2,4-dimethylhept-1-ene	1.49	0.71	0.05	ns	0.01	.	ns

Only compounds with a relative abundance >0.05% are listed.

*Tentative identification based on MS library match and RI literature match.

**Standard error of the mean (SEM).

ns, No significant difference (P > 0.10).

**Figure 2:**
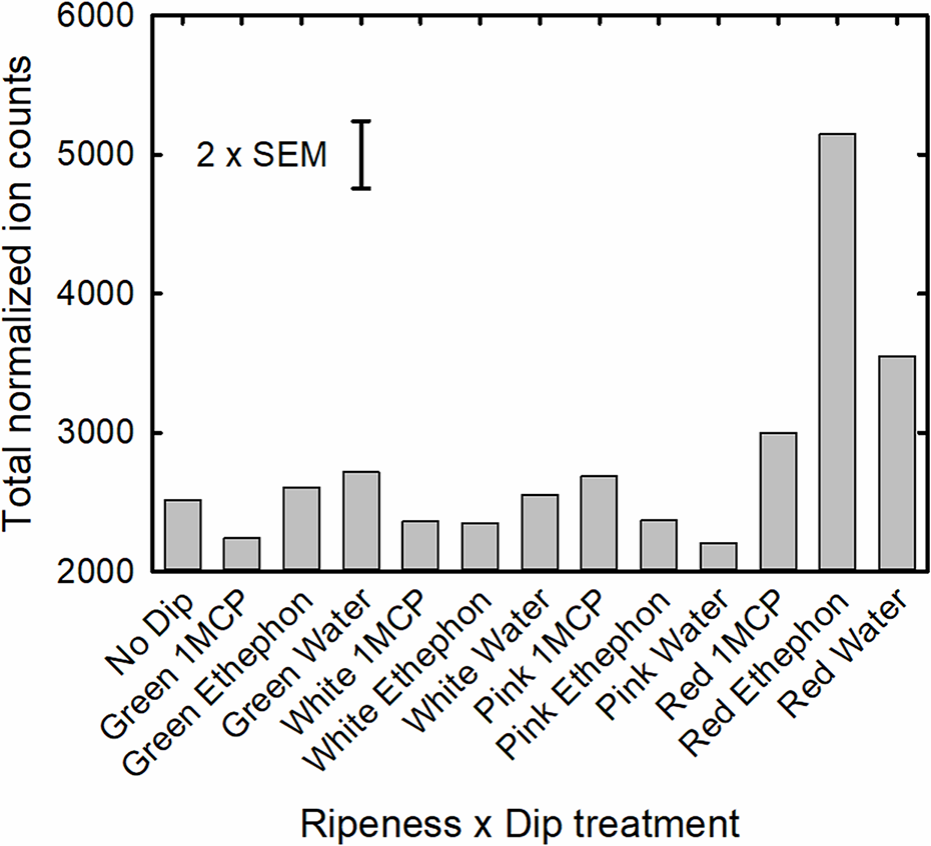
Total normalized ion counts of all volatile compounds of the ripeness x dip treatment interaction.

Total normalized ion counts of all volatile compounds were significantly affected by both stage and dip treatment, as well as a significant interaction of these two factors (**Table 5** and **Figure 2**). While total volatile concentrations of fruit treated at the green, white and pink maturity stages were similar, those treated at the red stage were significantly greater. Fruit treated at the red stage ripened 1 day longer than fruit treated at the other stages or the no dip control, which were all harvested on the day they turned fully red. As a result, the total concentration results of both straight-chain and branched-chain esters of red-treated fruit were more than twice that of fruit treated at other stages, with the concentration of ethyl 3-methylbutanoate, ethyl acetate, butyl butanoate and ethyl butanoate being 31-, 13-, 8- and 6-fold greater, respectively (**Supplementary Table S3**). In addition, concentrations of the important aroma compounds 4-methoxy-2,5-dimethylfuran-3-one, 5-hexyloxolan-2-one, heptan-2-one, hexanoic acid and (*E*)-hex-2-enal were 140%, 200%, 150%, 70%, and 25% greater in the red-treated fruit, respectively, than fruit treated at other stages. Similarly, total concentration of monoterpenoids was 67% greater in red-treated fruit. In contrast, the stage of treatment had little or no effect on fruit alcohol, ketone, norisoprenoid or hydrocarbon concentrations.

Treatments with ethephon and 1-MCP on green, white or pink fruit had little effect on volatile composition when fruit were red ripe. However, when red fruit were subjected to these treatments, total volatile concentrations in fruit treated with ethephon were 72% and 45% greater than those dipped in Harvista or water, respectively (**Figure 2**). This treatment effect on volatile concentration was primarily reflected in a significant treatment effect on straight-chain esters (P < 0.001), with those most affected being ethyl and acetate esters. Concentrations of ethyl hexanoate, ethyl butanoate, butyl acetate, and ethyl acetate were, 218%, 136%, 130%, and 93% greater in red, ethephon-treated fruit than in control fruit, respectively. In contrast, 1-MCP reduced the concentration of these volatiles. Ethyl hexanoate, ethyl butanoate, butyl acetate, and ethyl acetate concentration of red, 1-MCP treated fruit were 79%, 31%, 49%, and 3% of that of control fruit, respectively. Other volatile compounds that were strongly affected by the dip treatments (P < 0.001) included the branched chain esters 3-methylbutyl acetate and benzyl acetate. Other chemical classes of volatiles were not significantly affected by dip treatments except for alcohols (P=0.064), where ethephon enhanced concentrations of 2-ethylhexan-1-ol, and sesquiterpenoids (P=0.071), where ethephon enhanced concentrations of 3,7,11-trimethyldodeca-1,6,10-trien-3-ol (β-nerolidol), (3S,6S)-2,2,6-trimethyl-6-[(1S)-4-methylcyclohex-3-en-1-yl]oxan-3-ol (α-bisabolol oxide), and 1-methyl-4-[(2E)-6-methylhepta-2,5-dien-2-yl]cyclohexene (α-bisabolene).

**Figure 3:**
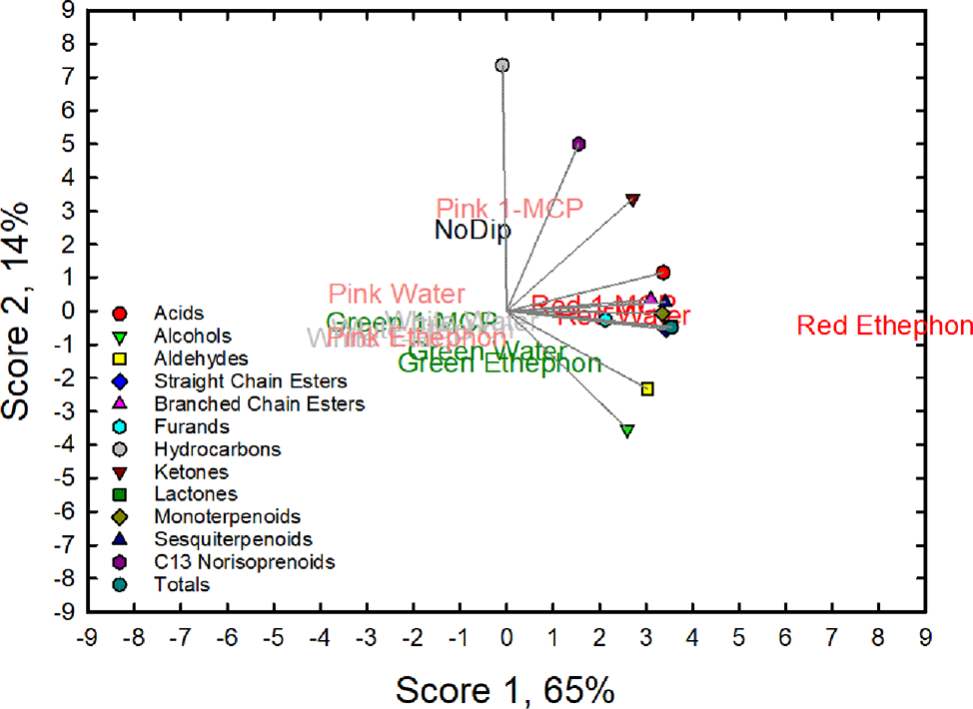
Principal component analysis (PCA) biplot for the first two principal component (PC) scores for 13 variables (acids, alcohols, aldehydes, straight-chain esters, branched-chain esters, furans, hydrocarbons, ketones, lactones, monoterpenoids, sesquiterpenoids, C13 norisoprenoid and totals) observed in ripe ‘Albion’ strawberry fruit treated with Ethephon, 1-MCP or water plus an absolute control (No Dip) at four development stages (Green, White, Pink and Red).

The effects of treatments on fruit volatile composition can be further illustrated by the score plot of the PCA analysis of chemical classes of the stage × dip interaction (**Figure 3**). Scores 1 and 2 accounted for 79% of the total variation with score one accounting for 65%. The volatile composition of the red-treated fruit had high score 1 values and was associated with high values of lactones, straight chain esters, sesquiterpenes, monoterpenes, and acids. Red fruit treated with ethephon (Red ethephon) had the highest score 1 value.

To explore the relationship of free amino acids with fruit volatiles, correlations were generated through PCA analysis (**Table 6**). A total of 41 volatile compounds had significant correlations with 14 amino acids. Aspartate, glutamine and glycine correlated with esters, furans, lactones and terpenoids. Methionine, phenylalanine, serine, and threonine correlated with sesquiterpenoids. Isoluecine and phenylalanine had a positive correlation with aldehydes, but a negative correlation was seen between isoleucine and tryptophan and 3-hexenal. Negative correlations were also observed between phenylalanine and tryptophan and the monoterpenoid terpinolene.

**Table 6:** P values of correlations between volatile compounds and free amino acids in ripe strawberry fruit that had been treated with Ethephon, 1-MCP, or water when fruit were green, white, pink or red.

Compound	Chemical class	Arg	Asp	Glu	Gln	Gly	Ile	Leu	Lys	Met	Phe	Ser	Trp	Thr	Tyr
Hexanoic_acid	Acid	ns	0.094	ns	ns	ns	ns	ns	ns	ns	ns	ns	ns	ns	ns
2-Ethyl-1-hexanol	Alcohol	ns	ns	ns	ns	ns	ns	ns	ns	ns	0.002	ns	ns	ns	ns
2-Methyl-2-propanol	Alcohol	ns	ns	ns	ns	ns	ns	0.001	ns	0.046	ns	ns	ns	ns	ns
(E)2-Octenal	Aldehyde	ns	ns	ns	ns	ns	ns	ns	ns	ns	0.079	ns	ns	ns	ns
3-Hexenal	Aldehyde	ns	ns	ns	ns	ns	0.013*	ns	ns	ns	ns	ns	0.086*	ns	ns
Benzaldehyde	Aldehyde	ns	ns	ns	ns	ns	ns	ns	ns	ns	0.028	ns	ns	ns	ns
3-Methyl butanal	Aldehyde	ns	ns	ns	ns	ns	0.029	ns	ns	ns	0.049	ns	0.005	ns	ns
Heptanal	Aldehyde	ns	ns	ns	ns	ns	0.037	ns	ns	ns	0.022	ns	ns	ns	ns
Nonanal	Aldehyde	ns	ns	ns	ns	ns	0.083	ns	ns	ns	0.019	ns	ns	ns	ns
Octanal	Aldehyde	ns	ns	ns	ns	ns	0.083	ns	ns	ns	0.008	ns	ns	ns	ns
Pentanal	Aldehyde	ns	ns	ns	ns	ns	ns	ns	ns	ns	0.061	ns	0.100	ns	ns
3-Methylbutyl acetate	Ester, branched chain	ns	0.084	ns	ns	ns	ns	ns	ns	ns	ns	ns	ns	ns	ns
Benzyl acetate	Ester, branched chain	0.094	0.043	ns	0.063	0.090	ns	ns	ns	ns	0.106	ns	ns	0.092	ns
Butyl acetate	Ester, straight chain	ns	0.054	ns	ns	ns	ns	ns	ns	ns	ns	ns	ns	ns	ns
Hexyl acetate	Ester, straight chain	0.120	0.020	ns	0.059	0.076	ns	ns	ns	ns	ns	ns	ns	0.079	ns
(Z)-Hex-2-enyl acetate	Ester,straight chain	0.052	0.009	ns	0.044	0.047	ns	ns	ns	ns	ns	ns	ns	0.032	ns
Butyl acetate	Ester,straight chain	ns	0.063	ns	ns	ns	ns	ns	ns	ns	ns	ns	ns	ns	ns
Ethyl butanoate	Ester,straight chain	ns	0.074	ns	ns	ns	ns	ns	ns	ns	ns	ns	ns	ns	ns
Methyl butanoate	Ester,straight chain	ns	ns	ns	ns	ns	ns	ns	ns	0.045	ns	ns	ns	ns	ns
Octyl butanoate	Ester,straight chain	ns	0.008	ns	0.024	0.043	ns	ns	ns	ns	ns	ns	ns	0.057	ns
Ethyl hexanoate	Ester,straight chain	ns	0.092	ns	ns	ns	ns	ns	ns	ns	ns	ns	ns	ns	ns
2,5-Dimethyl-4-methoxy-3(2H)-furanone	Furan	ns	0.010	ns	0.039	0.065	ns	ns	ns	ns	0.072	ns	ns	0.075	ns
1-Penten-3-one	Ketone	ns	ns	ns	ns	ns	ns	ns	ns	ns	0.024	ns	0.068	ns	ns
2-Heptanone	Ketone	ns	0.051	ns	ns	ns	ns	ns	ns	0.100	ns	ns	ns	ns	ns
2-Pentanone	Ketone	ns		ns	ns	ns	ns	ns	ns	0.096	ns	ns	ns	ns	ns
6-Methyl-5-heptene-2-one	Ketone	ns	0.070	ns	ns	ns	ns	ns	ns	ns	ns	ns	ns	ns	ns
1,2,4-Trimethyl-3-nitrobicyclo[3.3.1]nonan-9-one	Ketone	ns	0.023	ns	0.051	0.100	ns	ns	ns	ns	ns	ns	ns	ns	ns
γ-Decalactone	Lactone	ns	0.017	ns	0.055	0.106	ns	ns	ns	0.110	ns	ns	ns	ns	ns
γ-Dodecalactone	Lactone	ns	0.012	ns	0.101	0.156	ns	ns	ns	ns	ns	ns	ns	ns	ns
(E)-Linalool oxide	Monoterpenoid	ns	ns	ns	ns	ns	ns	0.054	ns	ns	0.113*	ns	ns	ns	ns
Terpinolene	Monoterpenoid	ns	0.048	ns	ns	ns	ns	ns	0.008	ns	0.073*	ns	0.072*	ns	0.018
α-Myrcene	Monoterpenoid	ns	0.054	ns	0.065	ns	ns	ns	ns	ns		ns	ns		ns
α-Terpineol	Monoterpenoid	ns	0.022	ns	0.021	0.058	ns	ns	ns	0.090	0.069	ns	ns	0.077	ns
Linalool	Monoterpenoid	ns	0.044	ns	0.048	0.100	ns	ns	ns	ns	ns	ns	ns	ns	ns
(Z)-Linalool oxide	Monoterpenoid	ns	0.059	ns	0.102	ns	ns	ns	ns	ns	ns	ns	ns	ns	ns
(-)-β-Pinene	Monoterpenoid	ns	ns	0.009	ns	ns	ns	ns	ns	0.091	ns	ns	ns	ns	ns
α-Bisabolol oxide	Sesquiterpenoid	ns	0.074	ns	0.110	ns	ns	ns	ns	ns	ns	ns	ns	ns	ns
α-Farnesene	Sesquiterpenoid	ns	0.013	ns	0.009	0.026	ns	ns	ns	0.018	0.056	0.103	ns	0.056	ns
(E)α-Famesene	Sesquiterpenoid	ns	0.021	ns	0.021	0.040	ns	ns	ns	0.021	0.085	0.098	ns	0.096	ns
(Z,E)-α-Farnesene	Sesquiterpenoid	ns	0.008	ns	0.012	0.026	ns	ns	ns	0.015		0.054	ns	0.081	ns
α-Bisabolene	Sesquiterpenoid	ns	0.013	ns	0.018	0.029	ns	ns	ns	0.023	0.050	0.050	ns	0.067	ns

Correlation values associated with P values are 0.476 (0.10), 0.539 (0.05) and 0.684 (0.001).

*Negative correlation.

ns, No significant difference (P > 0.10).

## Discussion

### Ethylene Effect on Strawberry Fruit Ripening

In this study, ethylene did not appear to play a major role in initiating or stimulating the ripening of immature strawberry fruit. Traditional ripeness indices, including red color development, sugar accumulation, acid loss and volatile synthesis were not affected by exogenous ethylene or 1-MCP treatments of immature fruit on the plant. This lack of treatment effect suggests that regulation of the normal ripening process in strawberry fruit was not affected by these one-time exogenous treatments and indicates that ethylene does not play a major role during normal development, maturation and ripening of strawberry fruit.

In contrast, some previous studies have reported that ethylene stimulated some aspects of postharvest ripening of harvested immature strawberry fruit. Ethylene treatments in the form of propylene or exogenous treatments with the ethylene precursor l-aminocyclopropane-1-carboxylic acid (ACC) stimulated fresh weight gain and color development in harvested green but not white fruit held in a sucrose solution (Perkins-Veazie et al., 1996). In pink harvested fruit, continuous exposure of fruit to 40 to 100 µl L^-1^ ethylene for 3 days at 20°C increased color development and softing, but not soluble solids, and 1-MCP reduced these effects (Tian et al., 2000). Villarreal et al. (2010; 2016) found ethephon increased anthocyanins and total sugars in harvested white fruit after 2 days at 22°C, but had no effect on titratable acids and treatment of fruit with 1-MCP inhibited these effects (Villarreal et al., 2010; Villarreal et al., 2016). Lopes et al. (2015) also reported that ethylene treatments increased total sugar content in white as well as green detached strawberry fruit. In addition, Tosseti et al. (2020) reported the propensity of ethylene to elicit an accumulation of reducing sugars, together with a concomitant decline in sucrose in cold stored strawberry. These postharvest sugar increases can not be attributed to normal ripening processes that occur on the plant, where sugar increase is attributed to import of sucrose from the leaves. In detached fruit, increase of sugars may come from degradation of cell wall components, since strawberry fruit do not accumulate or degrade starch during development, and sucrose can no longer be translocated into the fruit (Lopes et al., 2015). Treatment of harvested strawberry fruit with ethephon increased the expression the cell wall degrading enzyme polygalactronase (PG), which could release sugars, while treatments with 1-MCP decreased PG activity and fruit softing (Jiang et al., 2001; Villarreal et al., 2009).

These reports of ethylene stimulating aspects of fruit ripening may be a stress response of the fruit as a result of detatchment from the plant, which could alter fruit physiology. Perkins-Veazie (1996) found that ethylene production of white and red strawberry fruit increased 50 to 400% as a result of harvesting the fruit. However, in the present study, fruit remained on the plant and were not subjected to the stress of removal. Another possible response to detachment is the increase in phenolic compound content that was stimulated by ethylene treatment in detached green and white strawberry fruit (Lopes et al., 2015; Ayub et al., 2018b), while 1-MCP treatment reduced phenolic content (Villarreal et al., 2010). Treatment of fruit *in situ* in the present study had no effect on total phenolics regardless of the developmental stage at which the treatment was applied. The lack of ethylene and 1-MCP treatment effects on ripening suggests a control mechanism in the strawberry plant may mitigate any possible effects of these exogenous treatments. In addition, treatment effects on fruit composition were not measured until fruit were fully red ripe. Therefore, if there were any transient effects of the ethephon or 1-MCP treatment, they may no longer be apparent when fruit were harvested and analyzed, which occurred about 13, 6 or 4 days after treatment for fruit treated at the green, white or pink developmental stages, respectively. In a study that used transgenic strawberry plants that were partially insensitive to ethylene, white fruit had lower acid content compared to control fruit, but when fruit were red ripe these differences were no longer observed (Merchante et al., 2013). Another possible explaination for the lack of effect of the ethephon treatment is that commercial formulations of ethephon are extremely acidic (pH 2.0) and the pH of the strawberry fruit may be too low to effectively convert the ethephon to ethylene (Perkins-Veazie et al., 1996). However, this did not appear to be an issue in other studies where strawberry and other fruit were treated with ethephon (Singh and Janes, 2001; Villarreal et al., 2010; Zhang et al., 2014).

### Ethylene Effects on Fruit Development

Ethephon treatments did appear to affect specific aspects of fruit development, which were dependent on developmental stage of the fruit. In this study, treatment of green fruit with ethephon resulted in ripe fruit with greater mass and diameter than fruit treated at other stages or controls. In contrast, ethephon treatment of pink fruit resulted in ripe fruit that were smaller, firmer and had less mass. As well, ethephon treatment of white fruit resulted in red fruit with higher anthocyanin content. Responses of detached fruit to ethylene was also shown to be maturity dependent. As described above, Perkins-Veazie (1996) reported ethylene treatments increased fresh weight gain and color development in green but not white fruit. Balogh et al. (2005) looked at the effect of 1-MCP on expression of ripening-induced and ethylene-regulated genes and found the proportion of clones affected by 1-MCP was greatest in green fruit compared to that in white, pink or red fruit (Balogh et al., 2005). In transgenic plants that had reduced sensitivity to ethylene, white fruit had reduced concentration of tricarboxylic acid cycle intermediates including citric, succinic and malic acids, while red fruit had reduced sugars and the amino acids serine, phenylalanine and alanine (Merchante et al., 2013). Tian et al. (2000) suggested that fruit at different ripening stages may have different thresholds to ethylene (Tian et al., 2000).

### Ethylene Effects on Free Amino Acids and Volatiles

Free amino acid content of the fruit was also affected by the ethephon treatment and this effect appeared to be strongest in fruit that were treated with ethephon at the green and white developmental stage. Higher phenylalanine and tryptophan content in fruit treated with ethylene at the white development stage agrees with the higher anthocyanin content observed in ripe ‘Albion’ strawberry fruit. These aromatic amino acids are known to be the precursors for the biosynthesis of anthocyanins and flavonoids through the phenylpropanoid pathway (Zhang et al., 2011). This suggests a possible role of ethylene in strawberry anthocyanin formation. In addition studies indicate that phenylanine is an important precursor of aldehydes in the aroma volatile pathway (Kaminaga et al., 2006; Tieman et al., 2006; Gonda et al., 2010; Pang et al., 2012), in the present study a positive correlation was detected between these metabolites. Green and white fruit treated with ethephon also increased alanine content, which could reflect an increase in the alanine precursor pyruvate. Alanine was reported as an amino acid that changes during strawberry ripening (Pérez et al., 1992) and might serve as a precursor of ethyl esters, which are important volatile compounds in strawberry fruit aroma (Perez et al., 2002). According to Moing et al. (2001), the most aromatic strawberry cultivars also had the highest alanine concentration during maturation; however, in the present study no correlation was found between free alanine content and volatile compounds produced by the fruit. The high concentration of isoleucine and valine induced by ethephon application can also alter the volatile compound compostion. These branched amino acids can be transaminated and decarboxylated producing aldehydes, alcohols and esters (Gonda et al., 2010; Chen et al., 2016). However, there was only a positive correlation between isoleucine and aldehydes. The correlation between amino acids and terpenoids is poorly understood and future studies related to these pathways should be performed.

The production of aroma volatiles by red strawberry fruit was not affected by ethylene treatments of immature green, white or pink fruit. However, when red fruit were treated with ethephon an increase in volatile production was observed. It appears that when the fruit reached the red stage of ripeness, it developed responsiveness to ethylene, which primarily stimulated the production of ethyl and acetate esters. This response was inhibited by 1-MCP suggesting ethylene receptors were involved in this response. Other studies have reported that aroma volatile synthesis does not occur prior to color formation (Miszczak et al., 1995). The ethylene-enhanced esters included ethyl hexanoate, ethyl butanoate, butyl acetate, ethyl acetate, 3-methylbutyl acetate and benzyl acetate all of which have been reported to be important contributors to strawberry aroma (Forney et al., 2000; Aharoni, 2002; Vandendriessche et al., 2013). Other volatile compounds that contribute to the fruit aroma increased substancially with the additional day of ripening (red +24 h), but were not affected by the ethephon treatment.

The enhancement of ethyl and acetate ester synthesis induced by ethephon treatment could reflect a stress response of the strawberry fruit. During normal ripening on the plant, methyl butanoate and methyl hexanoate production in fruit of five strawberry cultivars increased 6- to 20-fold as fruit changed from 50% red to full red plus 24 h, while ethyl butanoate and ethyl hexanoate increased less than 50% (Forney et al., 2000). However, during postharvest ripening of ‘Kent’ strawberry fruit, the ratio of ethyl to methyl esters increased and in red fruit held for 4 days at 15°C, ethyl esters increased from 7% to 44% of the total volatiles (Miszczak et al., 1995). As well, ethyl esters were the dominate aroma volatile produced during postharvest ripening of white fruit, in contrast to the dominance of methyl esters in fruit ripened on the plant. Other postharvest stresses have been reported to increase the synthesis of ethyl and acetate esters in strawberry fruit and include low O_2_ and high CO_2_ atmospheres (Ke et al., 1994; Larsen and Watkins, 1995), storage temperature (Forney et al., 2000), and light (Miszczak et al., 1995).

The enzyme alcohol acyltransferase (AAT) is reported to be primarily responsible for ester synthesis in strawberry fruit and substrate availability in the fruit influences the specific esters synthesized (Pérez et al., 1993). The activity of AAT was first detected when strawberry fruit turn pink and increases as the fruit ripens (Pérez et al., 1996). The enzyme alcohol dehydrogenase (ADH) supplies alcohols and aldehydes as substrates for ester synthesis and the specific activity of this enzyme was reported to contribute to the determination of strawberry ester composition (Mitchell and Jelenkovic, 1995).

In climacteric fruit, the increase in ethylene biosynthesis at the onset of ripening plays an important role as a modulator of multiple aspects of maturation including taste and aroma development (Giovannoni, 2004; Barry and Giovannoni, 2007). In apple fruit, treatment with ethylene-inhibitor, results in a reduction of volatile compounds (Lurie et al., 2002; Kondo et al., 2005) and the expression of genes related to volatile biosynthesis, which were up-regulated by ethylene treatment (Yang et al., 2016). In transgenic apple lines that suppressed ethylene biosynthesis, reductions in volatile concentrations (Schaffer et al., 2007) as well as the activity of the enzyme alcohol acyltransferase (AAT) (Defilippi et al., 2005) were observed. In a similar way, ethylene-suppressed melon lines failed to produce aroma volatiles due to the inhibition of most of the steps of the ester biosynthetic pathway (Flores et al., 2001) demonstrating ethylene’s influence on the production of volatile compounds in climacteric fruit.

In contrast, in nonclimacteric fruit, limited information is available about the role of ethylene in volatile production. In grapes, treatment with the ethylene-inhibitor 1-MCP was found to partially repress the ripening-induced expression of the VvADH2 gene that encodes an alcohol dehydrogenase (Tesnière et al., 2006). In strawberry, which is extensively used as a model for nonclimacteric ripening studies, postharvest ripening and flavour development did not respond to treatment with ethylene (Perkins-Veazie et al., 1996). In a more recent study, transcript analysis showed that the expression of a volatile-related gene was down-regulated in ethylene-supressed transgenic lines, suggesting a role for ethylene in production of strawberry aroma compounds (Merchante et al., 2013), but no volatile composition was evaluated, showing the need for more studies about the role of ethylene on nonclimacteric volatile compound composition.

In the same way, transient downregulation of the ethylene biosynthesis-related gene *FaSAMS1* and the signaling gene *FaCTR1* at white stage can inhibit fruit pigment formation, that interestingly can be partially recovered by ethephon treatment (Sun et al., 2013).

It is important to remember that in the present study, while ethylene treatment at the white development stage increased anthocyanin content in ripe strawberry fruit, the 1MCP treatment did not inhibit this response, suggesting that there was no inhibitory effect on strawberry ethylene receptors and that a different ethylene signal transduction in attached strawberry fruit may occur. For example, anthocyanin synthesis may be dependent on other plant signals and not only be coordinated by fruit ethylene receptors, since an alternative pathway for ethylene signal transduction has been suggested by Zhang et al. (2014).

## Conclusions

Our results showed that one-time exogenous ethylene or 1-MCP treatments are not able to change the rate of ripening of immature strawberry fruit when fruit mature and ripen on the plant. Consequently, no change in rate of ripening, nor difference in fruit color, titratable acidity, pH, soluble solids, total phenolics, sugars or organic acids of ripe fruit was observed as a result of ethephon or 1-MCP treatments of immature fruit. However, ethylene did appear to alter fruit physiology in specific ways that were dependent on the stage of fruit development. Ethephon treatment of green fruit increase the ripe fruit size, while treatment of pink fruit decreased fruit size and increased firmness. Ethephon treatment of white fruit increased anthocyanin content, but did not affect visual color of the fruit. Fruit free amino acid content was also increased by ethylene, which was most apparent when fruit were treated at the green and white developmental stages. Treatment of red fruit with ethephon increased concentrations of ethyl- and acetate-esters, which were reduced by the 1-MCP treatment, but had no effect on other important aroma compounds. The ethylene effect on ripe fruit volatile metabolism suggests that ethylene may impart a stress response in the fruit as opposed to stimulating normal fruit ripening.

## Data Availability Statement

All datasets presented in this study are included in the article/**Supplementary Material**.

## Author Contributions 

LR, MJ, KM and CF carried out the experiments. CF and SF conducted the statistical analysis of the data. LR, MJ, SF, CF and MS analysed the GC-MS results. LR, KM, SF, CF and MS analysed the UHPLC results. LR, CF, MS and RA wrote the manuscript. RA wrote and approved project funding. All authors contributed to the article and approved the submitted version.

## Funding

This work was supported by the CAPES (Brazilian Coordination of Improvement of Higher-Level Personnel), AAFC (Agriculture and Agri-Food Canada) and UEPG (State University of Ponta Grossa).

## Conflict of Interest

The authors declare that the research was conducted in the absence of any commercial or financial relationships that could be construed as a potential conflict of interest.

## Supplementary Material

Supplementary Table S1 Amino acid concentrations and statistical test (ANOVA).

Supplementary Table S2 Volatile compounds identified by SPME-GCxGC-MS TOF analyses in the headspace of homogenized ripe strawberry fruit.

Supplementary Table S3 Headspace volatile composition of ripe ‘Albion’ strawberry fruit treated with Ethephon, Harvista (1-MCP) or water plus an absolute control (No Dip) at four development stages (Green, White, Pink and Red) and analyzed by SPME-GCxGC-MS TOF. Values are the total ion counts for each compound normalized against an internal standard of deuterated 2-hexanone-d5 (n=4).

Supplementary Figure S1 Four developmental stages of Albion strawberry. A- green, B- white, C- pink and D- red.

## Abbreviations

CG, gas chromatography; PAL, phenylalanine ammonia lyase; PCA, principal component analysis; PM, modulation period; SEM, standard error of the mean; SPME-GC–MS, solid phase micro extraction-gas chromatography–mass spectroscopy; SS, soluble solids; TA, titratable acidity; UHPLC, ultra high performance liquid chromatography; 1-MCP, 1-methylcyclopropene.

## References

[B1] Aharoni A. (2002). Gene expression analysis of strawberry achene and receptacle maturation using DNA microarrays. J. Exp. Bot. 53, 2073–2087. 10.1093/jxb/erf026 12324531

[B2] Ayub R. A. Bosetto L. Galvão C. W. Etto R. M. Inaba J. Lopes P. Z. (2016). Abscisic acid involvement on expression of related gene and phytochemicals during ripening in strawberry fruit Fragaria × ananassa cv. Camino Real. Sci. Hortic. (Amsterdam) 203, 178–184. 10.1016/j.scienta.2016.03.026

[B3] Ayub R. A. Reis L. Bosetto L. Lopes P. Z. Galvão C. W. Etto R. M. (2018a). Brassinosteroid plays a role on pink stage for receptor and transcription factors involved in strawberry fruit ripening. Plant Growth Regul. 84, 159–167. 10.1007/s10725-017-0329-5

[B4] Ayub R. A. Reis L. Lopes P. Z. Bosetto L. (2018b). Ethylene and brassinosteroid effect on strawberry ripening after field spray Efeito do etileno e do brassinoestereoide no amadurecimento do morango após aplicação no campo. Rev. Bras. =Frutic. 40, 1–6. 10.1590/0100-29452018544

[B5] Balogh A. Koncz T. Tisza V. Kiss E. Heszky L. (2005). The effect of 1-MCP on the expression of several ripening-related genes in strawberries. HortScience 40 (7), 2088–2090. 10.21273/HORTSCI.40.7.2088

[B6] Bapat V. A. Trivedi P. K. Ghosh A. Sane V. A. Ganapathi T. R. Nath P. (2010). Ripening of fleshy fruit: Molecular insight and the role of ethylene. Biotechnol. Adv. 28, 94–107. 10.1016/j.biotechadv.2009.10.002 19850118

[B7] Barry C. Giovannoni J. J. (2007). Ethylene and Fruit Ripening. J. Plant Growth Regul. 26, 143–159. 10.1007/s00344-007-9002-y

[B8] Chen H. Cao S. Jin Y. Tang Y. Qi H. (2016). The Relationship between CmADHs and the Diversity of Volatile Organic Compounds of Three Aroma Types of Melon (Cucumis melo). Front. Physiol. 7, 254. 10.3389/fphys.2016.00254 27445845 PMC4923263

[B9] Defilippi B. G. Kader A. A. Dandekar A. M. (2005). Apple aroma: Alcohol acyltransferase, a rate limiting step for ester biosynthesis, is regulated by ethylene. Plant Sci. 168, 1199–1210. 10.1016/j.plantsci.2004.12.018

[B10] Elmi F. Pradas I. Tosetti R. Cools K. Terry L. A. (2017). Effect of ethylene on postharvest strawberry fruit tissue biochemistry. Acta Hortic. 1156, 667–672. 10.17660/ActaHortic.2017.1156.97

[B11] Fait A. Hanhineva K. Beleggia R. Dai N. Rogachev I. Nikiforova V. J. (2008). Reconfiguration of the Achene and Receptacle Metabolic Networks during Strawberry Fruit Development. Plant Physiol. 148, 730–750. 10.1104/pp.108.120691 18715960 PMC2556830

[B12] Flores F. Ben Amor M. Jones B. Pech J. C. Bouzayen M. Latché A. (2001). The use of ethylene-suppressed lines to assess differential sensitivity to ethylene of the various ripening pathways in Cantaloupe melons. Physiol. Plant 113, 128–133. 10.1034/j.1399-3054.2001.1130117.x

[B13] Forney C. F. Kalt W. Jordan M. A. (2000). The composition of strawberry aroma is influenced by cultivar, maturity, and storage. HortScience 35 (6), 1022–1026. 10.21273/HORTSCI.35.6.1022

[B14] Giovannoni J. J. (2004). Genetic regulation of fruit development and ripening. Plant Cell 16, S170–S180. 10.1105/tpc.019158 15010516 PMC2643394

[B15] Gonda I. Bar E. Portnoy V. Lev S. Burger J. Schaffer A. A. (2010). Branched-chain and aromatic amino acid catabolism into aroma volatiles in Cucumis melo L. fruit. J. Exp. Bot. 61 (4), 1111–1123. 10.1093/jxb/erp390 20065117 PMC2826658

[B16] Iannetta P. P. M. Laarhoven L. J. Medina-Escobar N. James E. K. McManus M. T. Davies H. V. (2006). Ethylene and carbon dioxide production by developing strawberries show a correlative pattern that is indicative of ripening climacteric fruit. Physiol. Plant 127, 247–259. 10.1111/j.1399-3054.2006.00656.x

[B17] Jia H.-F. Chai Y.-M. Li C.-L. Lu D. Luo J.-J. Qin L. (2011). Abscisic Acid Plays an Important Role in the Regulation of Strawberry Fruit Ripening. Plant Physiol. 157, 188–199. 10.1104/pp.111.177311 21734113 PMC3165869

[B18] Jia H. Wang Y. Sun M. Li B. Han Y. Zhao Y. (2013). Sucrose functions as a signal involved in the regulation of strawberry fruit development and ripening. New Phytol. 198 (2), 453–465. 10.1111/nph.12176 23425297 10.1111/nph.12176

[B19] Jia H. Jiu S. Zhang C. Wang C. Tariq P. Liu Z. (2016). Abscisic acid and sucrose regulate tomato and strawberry fruit ripening through the abscisic acid-stress-ripening transcription factor. Plant Biotechnol. J. 14, 2045–2065. 10.1111/pbi.12563 27005823 PMC5043491

[B20] Jiang Y. Joyce D. C. Terry L. A. (2001). 1-Methylcyclopropene treatment affects strawberry fruit decay. Postharv. Biol. Technol. 23, 227–232. 10.1016/S0925-5214(01)00123-5

[B21] Kaminaga Y. Schnepp J. Peel G. Kish C. M. Ben-Nissan G. Weiss D. (2006). Plant phenylacetaldehyde synthase is a bifunctional homotetrameric enzyme that catalyzes phenylalanine decarboxylation and oxidation. J. Biol. Chem. 281, 23357–23366. 10.1074/jbc.M602708200 16766535

[B22] Ke D. Zhou L. Kader A. A. (1994). Mode of oxygen and carbon dioxide action on strawberry ester biosynthesis. J. Am. Soc. Hortic. Sci. 119 (5), 971–975. 10.21273/JASHS.119.5.971

[B23] Kondo S. Setha S. Rudell D. R. Buchanan D. A. Mattheis J. P. (2005). Aroma volatile biosynthesis in apples affected by 1-MCP and methyl jasmonate. Postharv. Biol. Technol. 36, 61–68. 10.1016/j.postharvbio.2004.11.005

[B24] Larsen M. Watkins C. B. (1995). Firmness and aroma composition of strawberries following short-term high carbon dioxide treatments. HortScience 30 (2), 303–305. 10.21273/HORTSCI.30.2.303

[B25] Lee J. Durst R. W. Wrolstad R. E. (2005). Determination of total monomeric anthocyanin pigment content of fruit juices, beverages, natural colorants, and wines by the pH differential method: Collaborative study. J. AOAC Int. 88, 1269–1278. 10.5555/jaoi.2005.88.5.1269 16385975

[B26] Lopes P. Z. Fornazzari I. M. Almeida A. T. Galvão C. W. Etto R. M. Inaba J. (2015). Effect of ethylene treatment on phytochemical and ethylene-related gene expression during ripening in strawberry fruit Fragaria x ananassa cv. Camino real. Genet. Mol. Res. 14, 16113–16125. 10.4238/2015.December.7.23 26662403

[B27] Lurie S. Pre-Aymard C. Ravid U. Larkov O. Fallik E. (2002). Effect of 1-methylcyclopropene on volatile emission and aroma in Cv. Anna apples. J. Agric. Food Chem. 50, 4251–4256. 10.1021/jf0200873 12105954

[B28] Merchante C. Vallarino J. G. Osorio S. Aragüez I. Villarreal N. Ariza M. T. (2013). Ethylene is involved in strawberry fruit ripening in an organ-specific manner. J. Exp. Bot. 64, 4421–4439. 10.1093/jxb/ert257 24098047 PMC3808323

[B29] Miszczak A. Forney C. F. Prange R. K. (1995). Development of aroma volatiles and color during postharvest ripening of Kent'strawberries. J. Am. Soc. Hortic. Sci. 120 (4), 650–655. 10.21273/JASHS.120.4.650

[B30] Mitchell W. C. Jelenkovic G. (1995). Characterizing NAD-and NADP-dependent alcohol dehydrogenase enzymes of strawberries. J. Am. Soc. Hortic. Sci. 120 (5), 798–801. 10.21273/JASHS.120.5.798

[B31] Moing A. Renaud C. Gaudillère M. Raymond P. Roudeillac P. Denoyes-Rothan B. (2001). Biochemical changes during fruit development of four strawberry cultivars. J. Am. Soc. Hortic Sci. 126 (4), 394–403. 10.21273/JASHS.126.4.394

[B32] Pang X. Guo X. Qin Z. Yao Y. Hu X. Wu J. (2012). Identification of aroma-active compounds in Jiashi muskmelon juice by GC-O-MS and OAV calculation. J. Agric. Food Chem. 60 (17), 4179–4185. 10.1021/jf300149m 22497266

[B33] Pérez A. G. Rios J. J. Sanz C. Olías J. M. (1992). Aroma Components and Free Amino Acids in Strawberry Variety Chandler during Ripening. J. Agric. Food Chem. 40, 2232–2235. 10.1021/jf00023a036

[B34] Pérez A. G. Sanz C. Olías J. M. (1993). Partial Purification and Some Properties of Alcohol Acyltransferase from Strawberry Fruits. J. Agric. Food Chem. 41, 1462–1466. 10.1021/jf00033a021

[B35] Pérez A. G. Sanz C. Olías R. Ríos J. J. Olías J. M. (1996). Evolution of Strawberry Alcohol Acyltransferase Activity during Fruit Development and Storage. J. Agric. Food Chem. 44, 3286–3290. 10.1021/jf960040f

[B36] Perez A. G. Rios J. J. Sanz C. Olias J. M. Pqez A. G. Rios J. J. (2002). Aroma components and free amino acids in strawberry variety Chandler during ripening. J. Agric. Food Chem. 40, 2232–2235. 10.1021/jf00023a036

[B37] Perkins-Veazie P. M. Huber D. J. Brecht J. K. (1996). In vitro growth and ripening of strawberry fruit in the presence of ACC, STS or propylene. Ann. Appl. Biol. 128, 105–116. 10.1111/j.1744-7348.1996.tb07094.x

[B38] Schaffer R. J. Friel E. N. Souleyre E. J. F. Bolitho K. Thodey K. Ledger S. (2007). A genomics approach reveals that aroma production in apple is controlled by ethylene predominantly at the final step in each biosynthetic pathway. Plant Physiol. 144, 1899–1912. 10.1104/pp.106.093765 17556515 PMC1949883

[B39] Singh Z. Janes J. (2001). Effects of postharvest application of ethephon on fruit ripening, quality and shelf life of mango under modified atmosphere packaging. Acta Hortic. (Int. Soc. Hortic. Sci.) 553, 599–602. 10.17660/ActaHortic.2001.553.141

[B40] Singleton V. L. Rossi J. A. Jr. Rossi J. A. Jr. (1965). Colorimetry of Total Phenolics with Phosphomolybdic-Phosphotungstic Acid Reagents. Am. J. Enol. Vitic. 16, 144–158. 10.12691/ijebb-2-1-5

[B41] Sun J.-H. Luo J.-J. Tian L. Li C.-L. Xing Y. Shen Y.-Y. (2013). New Evidence for the Role of Ethylene in Strawberry Fruit Ripening. J. Plant Growth Regul. 32, 461–470. 10.1007/s00344-012-9312-6

[B42] Symons G. M. Chua Y.-J. Ross J. Quittenden L. Davies N. Reid J. (2012). Hormonal changes during non-climacteric ripening in methylation. J. Exp. Bot. 63, 4741–4750. 10.1093/jxb/err147 22791823 PMC3428006

[B43] Tesnière C. Davies C. Sreekantan L. (2006). Analysis of the transcript levels of VvAdh1, VvAdh2 and VvGrip4, three genes highly expressed during Vitis vinifera L. berry development. Vitis-Geilweilerhof 45 (2), 75. 10.5073/vitis.2006.45.75-79

[B44] Tian M. S. Prakash S. Elgar H. J. Young H. Burmeister D. M. Ross G. S. (2000). Responses of strawberry fruit to 1-methylcyclopropene (1-MCP) and ethylene. Plant Growth Regul. 32, 83–90. 10.1023/A:1006409719333

[B45] Tieman D. Taylor M. Schauer N. Fernie A. R. Hanson A. D. Klee H. J. (2006). Tomato aromatic amino acid decarboxylases participate in synthesis of the flavor volatiles 2-phenylethanol and 2-phenylacetaldehyde. Proc. Natl. Acad. Sci. U. S. A. 103, 8287–8292. 10.1073/pnas.0602469103 16698923 PMC1472464

[B46] Tosetti R. Elmi F. Pradas I. Cools K. Terry L. A. (2020). Continuous Exposure to Ethylene Differentially Affects Senescence in Receptacle and Achene Tissues in Strawberry Fruit. Front. Plant Sci. 11, 174. 10.3389/fpls.2020.00174 32226433 PMC7080867

[B47] Trainotti L. Pavanello A. Casadoro G. (2005). Different ethylene receptors show an increased expression during the ripening of strawberries: Does such an increment imply a role for ethylene in the ripening of these non-climacteric fruits? J. Exp. Bot. 56, 2037–2046. 10.1093/jxb/eri202 15955790

[B48] Vandendriessche T. Vermeir S. Mayayo Martinez C. Hendrickx Y. Lammertyn J. Nicolaï B. M. (2013). Effect of ripening and inter-cultivar differences on strawberry quality. LWT Food Sci. Technol. 52, 62–70. 10.1016/j.lwt.2011.12.037

[B49] Villarreal N. M. Rosli H. G. Martínez G. A. Civello P. M. (2008). Polygalacturonase activity and expression of related genes during ripening of strawberry cultivars with contrasting fruit firmness. Postharv. Biol. Technol. 47, 141–150. 10.1016/j.postharvbio.2007.06.011

[B50] Villarreal N. M. Martínez G. A. Civello P. M. (2009). Influence of plant growth regulators on polygalacturonase expression in strawberry fruit. Plant Sci. 176, 749–757. 10.1016/j.plantsci.2009.02.019

[B51] Villarreal N. M. Bustamante C. A. Civello P. M. MartÃnez G. A. (2010). Effect of ethylene and 1-MCP treatments on strawberry fruit ripening. J. Sci. Food Agric. 90 (4), 683–689. 10.1002/jsfa.3868 20355099

[B52] Villarreal N. M. Marina M. Nardi C. F. Civello P. M. Martínez G. A. (2016). Novel insights of ethylene role in strawberry cell wall metabolism. Plant Sci. 252, 1–11. 10.1016/j.plantsci.2016.06.018 27717444

[B53] Yang X. Song J. Du L. Forney C. Campbell-Palmer L. Fillmore S. (2016). Ethylene and 1-MCP regulate major volatile biosynthetic pathways in apple fruit. Food Chem. 194, 325–336. 10.1016/j.foodchem.2015.08.018 26471562

[B54] Zhang J. Wang X. Yu O. Tang J. Gu X. Wan X. (2011). Metabolic profiling of strawberry (Fragaria×ananassa Duch.) during fruit development and maturation. J. Exp. Bot. 62, 1103–1118. 10.1093/jxb/erq343 21041374

[B55] Zhang J. Yu J. Wen C.-K. (2014). An alternate route of ethylene receptor signaling. Front. Plant Sci. 5, 1–6. 10.3389/fpls.2014.00648 PMC4238421 25477894

