# Supplementary figures and images for: Metabolic Profile of Strawberry Fruit Ripened on the Plant Following Treatment With an Ethylene Elicitor or Inhibitor
Source: Front Plant Sci. 2020 Jul 10;11:995. doi: 10.3389/fpls.2020.00995 (PMC7365940; doi:10.3389/fpls.2020.00995)

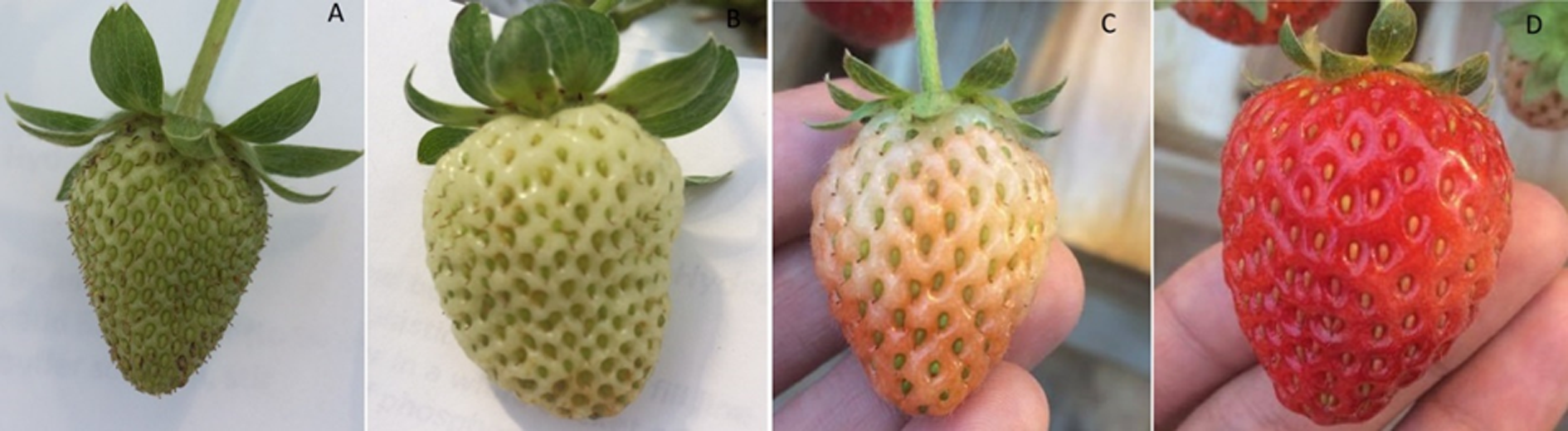

Supplement: Supplementary Figure S1 — Four developmental stages of Albion strawberry. A- green, B- white, C- pink and D- red. [file Image_1.tiff]
